# Supplementary material for: Pain Catastrophizing in Older Adults with Chronic Pain: The Mediator Effect of Mood Using a Path Analysis Approach
Source: J Clin Med. 2020 Jul 1;9(7):2073. doi: 10.3390/jcm9072073 (PMC7408783; doi:10.3390/jcm9072073)
Supplement: Supplementary file 1 [file jcm-09-02073-s001.pdf]

Supplementary Table 1. Correlation of psychometric scales

	ANX	DEP	PNI	ISI	PCS
ANX	1				
DEP	.732***	1			
PNI	.249***	.188***	1		
ISI	.297***	.275***	.177***	1	
PCS	.498***	.366***	.366***	.244***	1

***$P < .001$; ANX = anxiety as measured by General Well-being Schedule; DEP = depression as measured by General Well-being Schedule PNI =; Pain Intensity as measured by numeric rating scale for the previous seven days; ISI = Insomnia Severity Index; PCS = Pain Catastrophizing Scale.

Supplementary Table 2. Indirect effects upon pain catastrophizing (PCS)

	Estimate	Standardized regression coefficients bstd.(1)	Std. Err	z-value	p	Std.lv(2)
indir.WOM.to.PCS (thr. PNI)	0.459	0.013	0.214	2.144	0.032	0.459
indir.LAC.to.PCS (thr. PNI)	−0.680	−0.018	0.247	−2.759	0.003	−0.680
indir.COM.to.PCS (thr. PNI)	0.257	0.028	0.065	3.922	0.000	0.257
ind.WOM.to.PCS (thr. ISI)	0.270	0.010	0.165	1.636	0.102	0.270
indir.LAC.to.PCS (thr. ISI)	−0.081	−0.004	0.051	−1.603	0.114	−0.081
indir.COM.to.PCS (thr. ISI)	0.031	0.006	0.017	1.776	0.073	0.031
indir.ISI.to.PCS (thr. PNI)	0.095	0.012	0.051	1.874	0.061	0.095

Indir = indirect effect; PCS = Pain Catastrophizing Scale; WOM = Women; PNI = Pain Intensity as measured by numeric rating scale for the previous seven days; LAC = Low alcohol consumption; COM = Number of comorbidities; ISI = Insomnia Severity Index
(1) Completely standardized solution (estimates of parameters if the variances are unity).
(2) Dependent Variable is standardized.

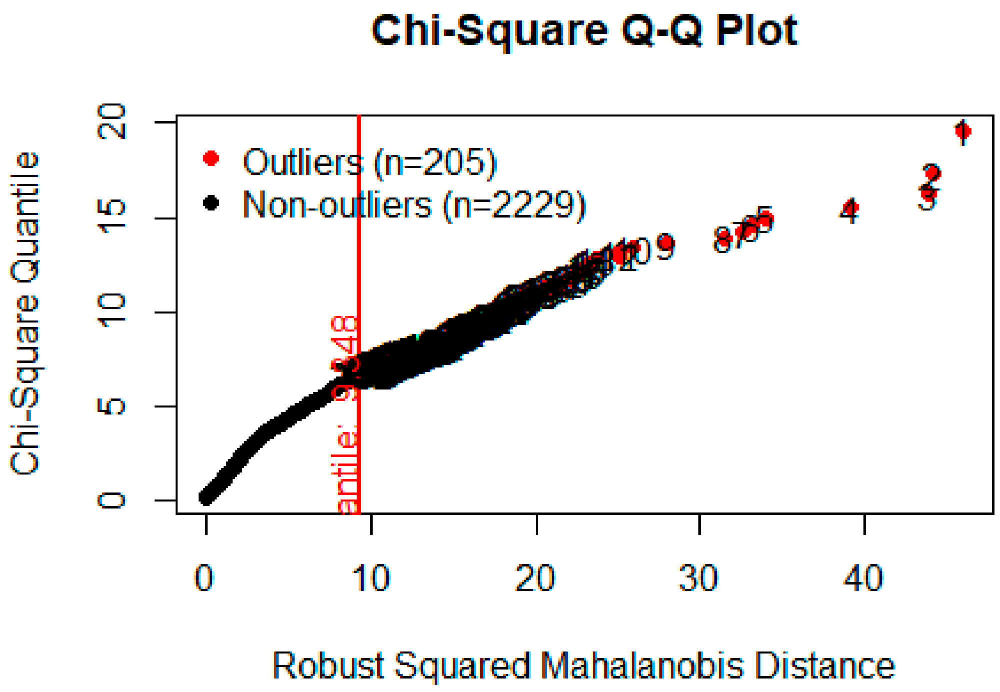

Supplementary Figure 1. The mahalanobis distance (MD) for all cases.
